# Analysis of TIR- and non-TIR-NBS-LRR disease resistance gene analogous in pepper: characterization, genetic variation, functional divergence and expression patterns

**DOI:** 10.1186/1471-2164-13-502

**Published:** 2012-09-21

**Authors:** Hongjian Wan, Wei Yuan, Qingjing Ye, Rongqing Wang, Meiying Ruan, Zhimiao Li, Guozhi Zhou, Zhuping Yao, Jing Zhao, Shujun Liu, Yuejian Yang

**Affiliations:** 1 Institute of Vegetables, Zhejiang Academy of Agricultural Sciences, Hangzhou, 310021, People’s Republic of China

## Abstract

**Background:**

Pepper (*Capsicum annuum* L.) is one of the most important vegetable crops worldwide. However, its yield and fruit quality can be severely threatened by several pathogens. The plant nucleotide-binding site (NBS)-leucine-rich repeat (LRR) gene family is the largest class of known disease resistance genes (R genes) effective against such pathogens. Therefore, the isolation and identification of such R gene homologues from pepper will provide a critical foundation for improving disease resistance breeding programs.

**Results:**

A total of 78 R gene analogues (CaRGAs) were identified in pepper by degenerate PCR amplification and database mining. Phylogenetic tree analysis of the deduced amino acid sequences for 51 of these CaRGAs with typically conserved motifs ( P-loop, kinase-2 and GLPL) along with some known R genes from *Arabidopsis* and tomato grouped these CaRGAs into the non-Toll interleukin-1 receptor (TIR)-NBS-LRR (CaRGAs I to IV) and TIR-NBS-LRR (CaRGAs V to VII) subfamilies. The presence of consensus motifs (i.e. P-loop, kinase-2 and hydrophobic domain) is typical of the non-TIR- and TIR-NBS-LRR gene subfamilies. This finding further supports the view that both subfamilies are widely distributed in dicot species. Functional divergence analysis provided strong statistical evidence of altered selective constraints during protein evolution between the two subfamilies. Thirteen critical amino acid sites involved in this divergence were also identified using DIVERGE version 2 software. Analyses of non-synonymous and synonymous substitutions per site showed that purifying selection can play a critical role in the evolutionary processes of non-TIR- and TIR-NBS-LRR RGAs in pepper. In addition, four specificity-determining positions were predicted to be responsible for functional specificity. qRT-PCR analysis showed that both salicylic and abscisic acids induce the expression of CaRGA genes, suggesting that they may primarily be involved in defence responses by activating signaling pathways.

**Conclusion:**

The identified CaRGAs are a valuable resource for discovering R genes and developing RGA molecular markers for genetic map construction. They will also be useful for improving disease resistance in pepper. The findings of this study provide a better understanding of the evolutionary mechanisms that drive the functional diversification of non-TIR- and TIR-NBS-LRR R genes in pepper.

## Background

Plant disease resistance genes (R genes) are important components of the genetic resistance mechanism in plants [1,2]. Over the past decade, several R genes conferring resistance to a wide spectrum of plant pathogens, including bacteria, fungi, oomycetes, viruses and nematodes, have been cloned from different plant species [2-5]. Sequence analyses revealed that these proteins share a high degree of homology and have a number of conserved motifs. These include a nucleotide-binding site (NBS), a leucine-rich repeat (LRR) region, a motif homologous to the cytoplasmic domains of the *Drosophila* Toll protein and the mammalian interleukin-1 receptor (TIR), a coiled-coil (CC) or leucine zipper structure, a transmembrane domain (TM) and a protein kinase domain [6]. Although a wide range of pathogens are involved, these R genes encode a limited set of proteins that can be classified into several superfamilies, including NBS-LRR, a receptor-like kinase, LRR-TM and TM-CC [2,7].

The NBS-LRR class of R genes can be divided into two subfamilies (TIR-NBS-LRR and non-TIR-NBS-LRR) based on the features of their N-terminal structure [2,8]. These two subfamilies can also be distinguished (95% accuracy) by the last residue, D (Aspartate) or W (Tryptophan), of the conserved kinase-2 motif within the NBS domain [9]. The former corresponds to the TIR-NBS-LRR subfamily, whereas the latter corresponds to the non-TIR-NBS-LRR subfamily of R genes. The ‘NBS’ and ‘LRR’ domains in the NBS-LRR R genes have different roles during host–pathogen recognition. The highly conserved NBS domains can bind and hydrolyze ATP or GTP [10], whereas the LRR motif is typically involved in protein–protein interactions and is responsible for recognition specificity [2,11,12].

To date, eight conserved motifs have been identified in the NBS domain of plant non-TIR- and TIR-NBS-LRR R genes, including P-loop, kinase-2, kinase-3a, GLPL, RNBS-A-TIR, RNBS-D-TIR, RNBS-A-non-TIR and RNBS-D-non-TIR [8]. The first four conserved motifs are common in the TIR and non-TIR-NBS-LRR subfamilies. The RNBS-A-TIR and RNBS-D-TIR motifs are specific to the TIR-NBS-LRR subfamily. The remaining two motifs, RNBS-A-non-TIR and RNBS-D-non-TIR, belong to the non-TIR-NBS-LRR subfamily. These highly conserved motifs within the NBS domain occur across different plant species, making it possible to isolate R gene analogues (RGAs) from other crops using degenerate polymerase chain reaction (PCR) [13-19]. At present, more than 1600 NBS-LRR-type RGAs have been amplified via PCR from a wide range of plant species, and they have been arranged in clusters similar to R genes in plant genomes [5,20]. Some of these are closely linked to known R gene loci or form a part of the R genes [21-23].

In recent years, the evolutionary patterns of NBS-LRR R genes have been investigated extensively in different plant species. For example, in annual species, such as *Arabidopsis* and rice, studies have shown that tandem and segmental gene duplication, gene conversion, unequal crossing-over, ectopic recombination and diversifying selection seem to be the primary evolutionary modes of NBS-LRR R genes [3,24-27]. In woody perennial species (e.g. grapevine and poplar), tandem gene duplication and recombination play major roles in NBS-LRR R gene expansion [28]. Point mutations, small insertions or deletions and gene loss have been proposed as the primary mechanisms by which NBS-LRR R genes evolve [29,30]. Therefore, the evolution of plant NBS-LRR R genes appears to be a complex process.

Pepper (*Capsicum annuum* L.), a member of the botanical family Solanaceae, is an important vegetable crop worldwide. However, its production is affected because it is prone to many diseases. At present, three R genes conferring resistance to strains of *Xanthomonas campestris pv. vesicatoria* and root-knot nematodes have been identified from pepper [31-33]. Of them, two genes (*Bs2* and *CaMi*) encode motifs characteristic of the NBS-LRR class of resistance genes. Moreover, some RGAs in pepper have been identified by modified amplified fragment length polymorphisms, NBS profiling and specific PCR amplification with primers designed from conserved regions of the NBS domain [34-37]. However, no detailed analysis of RGA characteristics is currently available. In this paper, we followed a PCR-based protocol using R gene-specific degenerate primers and data mining to identify and characterize the NBS-LRR CaRGAs and identify putative R genes in pepper. We also analyzed the genetic variations and phylogeny in pepper. Functional divergence analysis provided statistical evidence for altered selective constraints during protein evolution between the two subfamilies and identified some critical amino acid sites involved in this functional divergence. Analyses of non-synonymous (Ka) and synonymous (Ks) substitutions per site revealed a purifying selection in the evolutionary processes of non-TIR- and TIR-NBS-LRR CaRGAs in pepper. Several specificity-determining positions (SDPs) responsible for functional specificity were also predicted. Finally, the expression of representative CaRGAs was analysed in response to hormones and in different organs.

## Results and discussion

### Identification of non-TIR- and TIR-NBS-LRR CaRGAs in pepper

Candidate non-TIR- and TIR-NBS-LRR CaRGAs were identified in pepper using two approaches, PCR amplification with degenerate primers and database mining. Two pairs of degenerate primers, previously designed based on conserved domains (P-loop and GLPL regions) among known NBS-LRR R genes from other plant species [37-39], were used. Two bands of the predicted size (~500 bp) were amplified using the genomic DNA of pepper (Additional file 1). The bands were then excised from agarose gels and cloned. A total of fifty clones were randomly selected for sequencing, twenty-four of which were highly homologous to NBS-LRR sequences or known R genes from other plant species. These sequences were designated as NBS-LRR CaRGAs. The remaining clones were homologous to either a putative polyprotein or a hypothetical LRR protein. This finding suggests that degenerate PCR amplification is a very effective method for isolating potential CaRGAs from pepper.

A total of fifty-four CaRGAs were identified using the key word ‘*Capsicum* resistance gene’ in a search of the National Center for Biotechnology Information (NCBI) non-redundant protein database (http://www.ncbi.nlm.nih.gov/). A total of seventy-eight CaRGAs were obtained using these two methods. Among the seventy-eight CaRGAs, fifty-five had uninterrupted open reading frames (seventeen from amplified products, thirty-eight from data mining) and twenty-three CaRGAs had stop codons in the reading frames. These CaRGAs may be non-functional genes. Hence, they were excluded from further analysis. Moreover, two R genes reported previously were selected for further analysis [32,33]. In addition, more than one hundred and seventy and four hundred NBS-LRR R genes from *Arabidopsis* and rice [3,26], respectively, were used to retrieve potential R genes or CaRGAs from pepper. However, no novel CaRGAs or R genes were found. The nucleotide and amino acid sequences of all these pepper CaRGAs are listed in Additional file 2.

### Sequence analysis and phylogenetic relationship between non-TIR- and TIR-NBS-LRR RGAs

BLASTX searches revealed that the fifty-seven CaRGAs had a certain degree of identity with known R genes as well as some RGAs from other plant species. They contained the conserved NB-ARC (nucleotide-binding adaptor shared by *Apaf-1*, R proteins and *Ced-4*) domain of known R genes [40-42]. Further analysis indicated that these CaRGAs, except for CaRGA54 (AF513549), CaRGA55 (FJ605104), CaRGA56 (FJ605105) and CaRGA57 (FJ605107), also included typically conserved motifs such as P-loop, kinase-2 and GLPL.

**Figure 1:**
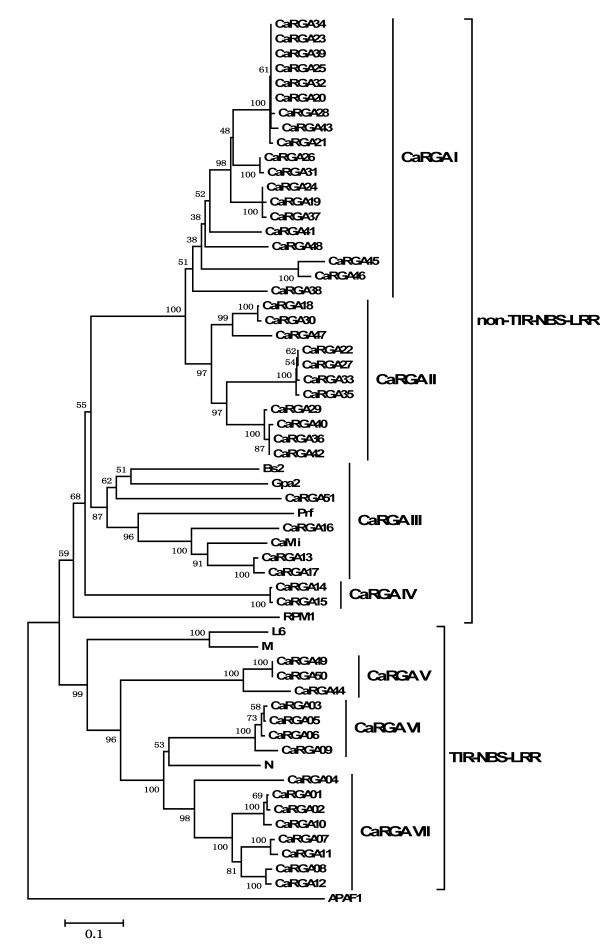
**Phylogenetic tree analysis based on alignment of the deduced amino acid sequences of pepper CaRGAs with known R genes. **The tree was constructed by the neighbor-joining method using MEGA 5.0 software. The 51 CaRGA sequences and 2 resistance genes were grouped into seven subgroups: I–VII. I–IV are part of the non-TIR-NBS-LRR family, whereas V–VII belong to the TIR-NBS-LRR family. Bootstrap values (1000 replicates) are given below the branches. The known R genes with the NBS domain that were used were *N *(U15605), *L6 *(U27081), *M *(U73916), *Prf *(U65391), *Gpa2 *(AF195939) and *RPM1 *(X87851).

To investigate the evolutionary relationships among the NBS-LRR CaRGAs of pepper, phylogenetic trees were constructed based on the region between the P-loop and GLPL motifs using Molecular Evolutionary Genetics Analysis (MEGA) 5.0 software [43]. The aforementioned four CaRGAs were excluded from subsequent analyses because they lacked conserved P-loop and GLPL motifs. Thus, a total of fifty-three CaRGAs of pepper were used for phylogenetic tree construction. Six known R genes (*RPM1*, *Gpa2*, *L6*, *M*, *N* and *Prf*), downloaded from the GenBank database, were also included in the analysis. The phylogenetic tree is shown in Figure 1. Two primary groups, non-TIR- and TIR-NBS-LRR, were obviously distinguished, which is consistent with previously described patterns [9,15]. The non-TIR-NBS-LRR subfamily included thirty-eight members (CaRGA13–CaRGA43, CaRGA45–CaRGA48, CaRGA51, *BS2* and *CaMi*), whereas only fifteen members (CaRGA01–CaRGA12, CaRGA49–CaRGA50 and CaRGA44) belonged to the TIR-NBS-LRR subfamily. These results support the view that both TIR-NBSLRR and non-TIR-NBS-LRR R genes occur in dicot species [15]. Within the non-TIR-NBS-LRR subfamily, CaRGAs could be separated into four subgroups, which were designated CaRGA I–IV. In the TIR-NBS-LRR subfamily, the CaRGAs could be divided into three subgroups, which were designated CaRGA V–VII. Each of these seven subgroups included different numbers of CaRGA members. The CaRGA III, CaRGA IV, CaRGA V and CaRGA VI subgroups contained two to four members, whereas the CaRGA I, CaRGA II and CaRGA VII subgroups consisted of eight to nineteen members. This result reflects a difference in the abundance of these RGA subgroups in the pepper genome (Figure 1). The characteristics of these non-TIR- and TIR-NBS CaRGA sequences from pepper are shown in Table 1.

**Table 1:** **Characteristics of non-TIR and TIR-NBS CaRGAs from pepper (*Capsicum anuum *** **L.)**

**Non-TIR/TIR**	**Class**	**Clone name**	**Length of encoding nucleotide** **residues (bp) and amino acids (aa)**	**GenBank accession number**
Non-TIR-NBS-LRR	CaRGA I	CaRGA23;CaRGA28;CaRGA34;CaRGA43; CaRGA25;CaRGA39;CaRGA32;CaRGA20; CaRGA21;CaRGA26;CaRGA31;CaRGA24; CaRGA19;CaRGA37;CaRGA48;CaRGA41; CaRGA45; CaRGA46; CaRGA38	723/240;722/240;722/240;706/234728/242;701/233;722/240;697/232 684/227;728/242;712/237;731/243 730/243;731/243;613/204;703/234 510/170; 608/202; 707/235	DQ205986;DQ205995; DQ206007; DQ206021; DQ205988;DQ206016; DQ206000; DQ205982; DQ205984;DQ205989; DQ205999; DQ205987; DQ205981;DQ206010; FJ605106; DQ206019; FJ605101; FJ605102; DQ206015
	CaRGA II	CaRGA18;CaRGA30;CaRGA47;CaRGA22; CaRGA27;CaRGA33;CaRGA35;CaRGA29; CaRGA36; CaRGA40; CaRGA42	704/234;706/234;628/209;725/241 709/236;692/230;714/238;655/218 725/241; 725/241;725/241	DQ205980; DQ205998; FJ605103; DQ205985; DQ205992; DQ206001;DQ206008; DQ205997; DQ206009; DQ206018; DQ206020
	CaRGA III	CaRGA51;CaRGA16;CaRGA13; CaRGA17 *Bs2*; *CaMi*	525/175;513/171;501/167; 501/167 2715/905; 3774/1257	AF513548; JN112315; JN112312; JN112316 AF202179; DQ465824
	CaRGA IV	CaRGA14; CaRGA15	516/172; 516/172	JN112313; JN112314
TIR-NBS-LRR	CaRGA V	CaRGA49; CaRGA50; CaRGA44	512/171;563/178; 492/161	FJ605108; FJ605109; FJ605100
	CaRGA VI	CaRGA03;CaRGA09;CaRGA05; CaRGA06	495/165;456/152; 495/165;495/165	JN112302; JN112308; JN112304; JN112305
	CaRGA VII	CaRGA04;CaRGA02;CaRGA10;CaRGA01; CaRGA07;CaRGA11;CaRGA08; CaRGA12	513/171;504/168;504/168;504/168 504/168;504/168;504/168;504/168	JN112303;JN112301;JN112309;JN112300; JN112306; JN112310; JN112307; JN112311

We further revealed that sequence identity between the CaRGAs from PCR amplification and database mining ranged from 21.9% (CaRGA III–V) to 64.9% (CaRGA I and CaRGA II) at the amino acid level (Table 2). This finding indicates that these CaRGAs are characterized by a high degree of divergence. There were also different degrees of variation in sequence homology within each group. The highest value was observed for CaRGA25 and CaRGA20 (99.8%) (CaRGA I) and the lowest value was observed for CaRGA17 and CaRGA51 (39.8%) (CaRGA III). CaRGA25 and CaRGA20 may have evolved from a common ancestor gene and CaRGA17 and CaRGA51 may have convergent evolutionary origins. Compared with known R genes, the sequence identity ranged from 18.5% (between *Gpa2* and CaRGA V) to 69.1% (between *N* and CaRGA VI) at the amino acid level (Table 2). We also analyzed sequence identity between the CaRGAs from PCR amplification and database mining and two R genes (*Bs2* and *CaMi*) reported. The highest value was observed for CaRGA13 and *CaMi* (80.7%) and the lowest value was observed for CaRGA44 and *CaMi* (20.3%). In addition, the BLASTP search of these seven subgroups revealed that their sequences had the highest degree of identity and similarity with known R genes or RGAs from Solanaceae crops. CaRGAs I and II showed the highest degree of similarity with the R3a-like disease-resistance protein gene from *Solanum demissum*. The sequences of CaRGAs III and VII were most similar to *Mi-1.4* from *Solanum* sp. VFNT and bacterial spot disease-resistance protein genes (*Bs4*) from *S. lycopersicum*, respectively. The remaining CaRGAs IV, V and VI were most similar to the NBS-encoding resistance protein genes from *S. aculeatissimum*, *S. circaeifolium* and *S. lycopersicum*, respectively (Table 3).

**Table 2:** Amino acid sequence similarity (%) among representatives of the seven CaRGA subgroups identified from pepper and six known NBS-LRR plant R genes

**Name**	**CaRGA I**	**CaRGA II**	**CaRGA III**	**CaRGA IV**	**CaRGA V**	**CaRGA VI**	**CaRGA VII**	***RPM1***	***Gpa2***	***L6***	***M***	***N***	***Prf***	***Bs2***	***CaMi***
CaRGA I (CaRGA23)	100														
CaRGA II (CaRGA33)	64.9	100													
CaRGA III (CaRGA13)	40.6	39.4	100												
CaRGA IV (CaRGA14)	36.3	33.9	38.2	100											
CaRGA V (CaRGA44)	22.2	22.2	21.9	23.0	100										
CaRGA VI (CaRGA05)	27.5	28.8	26.8	24.7	43.9	100									
CaRGA VII (CaRGA01)	27.3	29.8	23.4	25.8	47.5	61.2	100								
*RPM1*	29.9	31.1	33.3	33.9	21.7	24.4	24.5	100							
*Gpa2*	39.4	36.3	47.8	31.5	18.5	23.8	23.0	31.7	100						
*L6*	29.2	29.2	26.7	22.8	38.6	35.8	33.3	22.0	25.0	100					
*M*	29.2	29.8	28.0	25.3	40.9	35.0	34.3	24.6	28.8	81.8	100				
*N*	28.8	31.3	28.1	26.7	49.0	69.1	66.1	24.5	26.0	37.7	35.6	100			
*Prf*	33.3	33.3	53.0	34.5	18.4	23.6	21.5	26.5	47.2	23.5	24.1	25.0	100		
*Bs2*	38.5	34.8	42.3	32.7	27.0	27.3	26.9	28.9	51.9	26.2	27.5	26.1	40.5	100	
*CaMi*	45.3	47.2	80.7	37.2	20.3	23.9	24.4	31.5	42.9	21.9	22.8	28.0	50.0	40.5	100

The analysis was carried out with sequences spanning the P-loop and GLPL motifs.

**Table 3:** Sequence homology comparisons between representatives of the identified pepper CaRGAs subgroups and its closest homolog in the GenBank

Pepper RGAs*	**GenBank accessions**	**Plant species**	**Similar to**	**Length of BLASTX alignment**	**Identity (%)**	**Similarity (%)**	**E-value**
CaRGA I (CaRGA23)	EF613519	*Solanum demissum*	R3a-like disease resistance protein gene	171	82	93	1e-64
CaRGA II(CaRGA33)	EF613542	*Solanum demissum*	R3a-like disease resistance protein gene	171	77	87	5e-72
CaRGA III (CaRGA13)	DQ863287	*Solanum sp.* VFNT	Mi-1.4 disease resistance protein gene	166	83	91	4e-64
CaRGA IV (CaRGA14)	GU199034	*Solanum aculeatissimum*	NBS-encoding resistance protein gene (RGA8)	171	88	94	4e-85
CaRGA V (CaRGA44)	Y16680	*Solanum circaeifolium*	NBS-encoding resistance protein gene (clone crc Rgen8)	161	68	79	2e-39
CaRGA VI (CaRGA05)	AF404422	*Solanum Lycopersicon*	Nucleotide binding region of resistance-like gene (Q8)	165	84	90	3e-76
CaRGA VII (CaRGA01)	AY438027	*Solanum Lycopersicon*	Bacterial spot disease resistance protein gene (Bs4)	168	78	90	2e-65

*RGA I to RGA VII represent seven classes of the pepper CaRGAs, respectively.

### Multiple sequence alignments of non-TIR- and TIR-NBS-LRR RGAs in pepper

**Figure 2:**
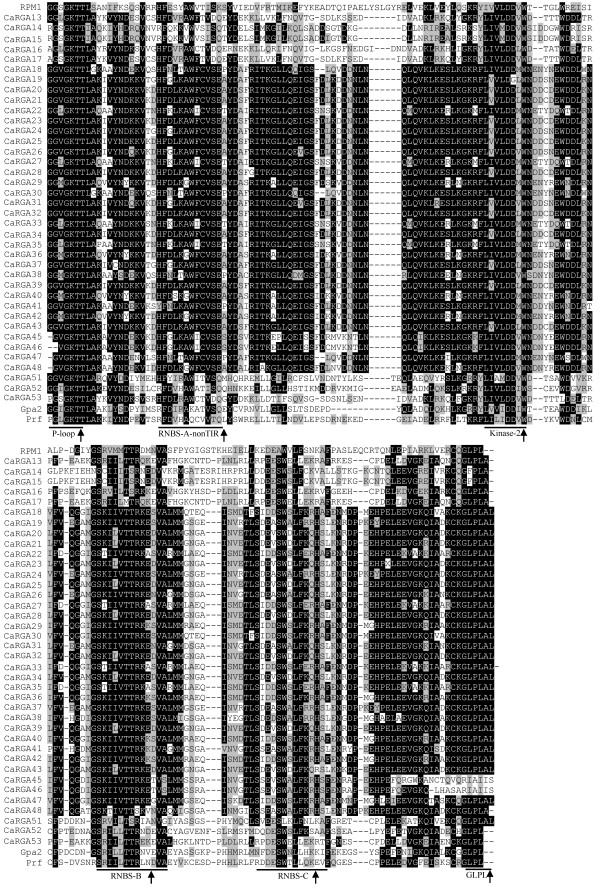
**Amino acid sequence alignment between P-loop and GLPL of non-TIR-NBS-LRR CaRGAs with the NBS domains of known R genes *Prf *(U65391), *Gpa2 *(AF195939) and *RPM1 *(X87851). **Conserved domains are highlighted and indicated by an arrow. The alignment was constructed using BioEdit 7.0.0 software. The threshold (%) for shading was set at 50. Similar amino acid residues are shaded grey and identical amino acid residues are shaded black.

**Figure 3:**
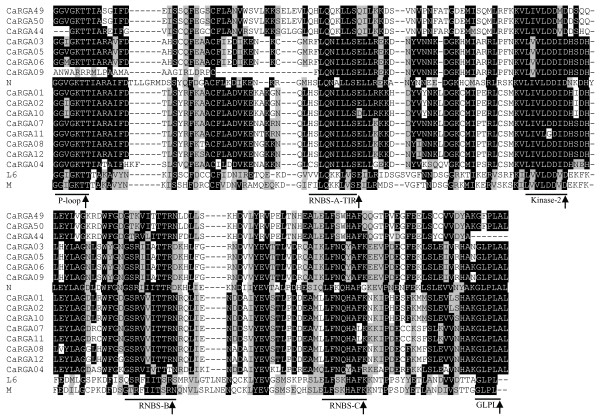
**Amino acid sequence alignment between P-loop and GLPL of TIR-NBS-LRR CaRGAs with the NBS domains of known R genes *N *(U15605), *L6 *(U27081) and *M *(U73916). **The conserved domains are highlighted and indicated with an arrow. The alignment was performed using BioEdit 7.0.0 software. The threshold (%) for shading was set at 50. Similar amino acid residues are shaded grey and identical amino acid residues are shaded black.

The CaRGAs identified from pepper were obviously separated into non-TIR- and TIR-NBS-LRR subfamilies (Figure 1). Consequently, multiple sequence alignments of non-TIR- and TIR-NBS-LRR RGAs were performed separately. The sequence alignment between non-TIR-NBS-LRR RGAs and known disease R genes, including *RPM1*, *Gpa2* and *Prf*, revealed six conserved motifs (i.e. P-loop, RNBS-A-non-TIR, kinase-2, RNBS-B, RNBS-C and GLPL) (Figure 2). Similarly, alignment analysis with known R genes *N*, *M* and *L6* revealed that the TIR-NBS-LRR RGAs also contain six conserved motifs (i.e. P-loop, RNBS-A-TIR, kinase-2, RNBS-B, RNBS-C and GLPL) (Figure 3). The presence of these consensus motifs within the non-TIR- and TIR-NBS-LRR CaRGAs provides further evidence that the cloned sequences are NBS-LRR gene family members.

### Analysis of functional divergence between the non-TIR- and TIR-NBS-LRR CaRGA subfamilies

Type I and II functional divergence between the non-TIR- and TIR-NBS-LRR subfamilies of pepper was assessed by posterior analysis using DIVERGE 2.0 software, which evaluates the (site-specific) shifted evolutionary rate after gene duplication or speciation [44,45]. Posterior analysis results in a site-specific profile for predicting important amino acid residues responsible for functional divergence. The estimation was based on multiple sequence alignments and a neighbor-joining (NJ) tree of the NBS domain of the pepper RGAs, with clear separation of the two different subfamilies, non-TIR- and TIR-NBS-LRR (Figure 1). The coefficient of type-I functional divergence (θ_I_) between the non-TIR- and TIR-NBS-LRR subfamilies was significantly greater than 0 (θ_I_ = 0.533 ± 0.156, *P* < 0.05). This result suggests that the altered functional constraint between the subfamilies is statistically significant and that some amino acid sites are subjected to different site-specific shifts in evolutionary rate that can lead to a subfamily-specific functional evolution after diversification. Compared with the findings for type-I functional divergence, the coefficient of type-II functional divergence (θ_II_) between the non-TIR- and TIR-NBS-LRR subfamilies was less than 0. Therefore, type-I functional divergence was the primary pattern for the evolution of the non-TIR- and TIR-NBS-LRR subfamilies in pepper.

We further estimated the critical amino acid residues responsible for the functional divergence by calculating the site-specific profile based on a posterior probability (*Q*_*k*_) analysis of the non-TIR- and TIR-NBS-LRR subfamilies. Among all of the aligned sites, the *Q*_*k*_ values of most sites were <0.5 (Additional file 3). To reduce false positives, *Q*_*k*_ > 0.70 was used as a cut-off to identify critical amino acid residues associated with type-I functional divergence between the non-TIR- and TIR-NBS-LRR subfamilies. A total of thirteen sites (positions 21, 22, 23, 69, 84, 104, 113, 116, 118, 125, 146, 155 and 165) were predicted (Additional file 3). Among these sites, the *Q*_*k*_ value of site 22 was 0.878, which was predicted to be highly related to functional divergence, whereas the degree of relation to functional divergence was lowest at site 155 (*Q*_*k*_ = 0.729).

**Figure 4:**
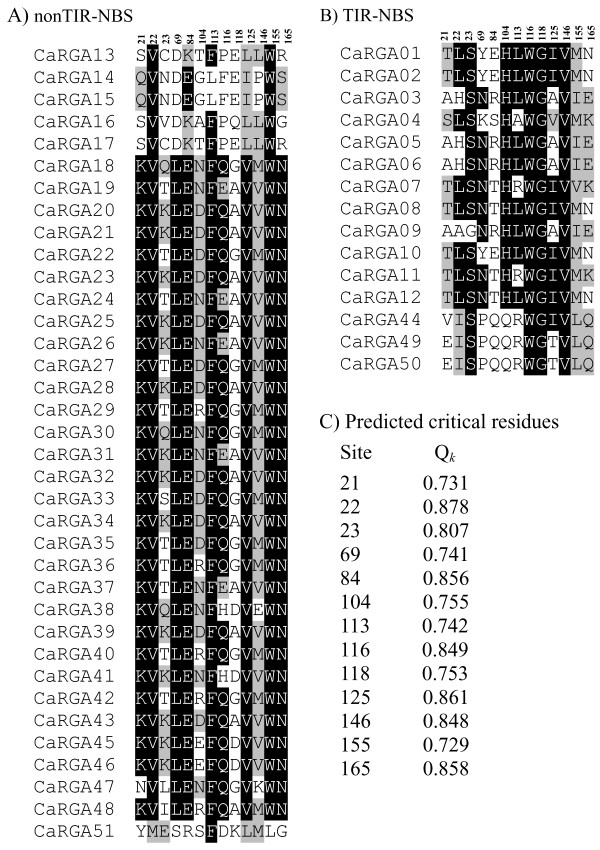
**Functional divergence-related amino acid site candidates (posterior probability, *Q***_**k **_**> 0.7). **A site-specific profile based on the posterior probability (*Q*_k _)was used to identify the critical amino acid sites responsible for the functional divergence between the non-TIR- and TIR-NBS RGA subfamilies. According to the definition, a large *Q*_k _value indicates a high possibility that the functional constraint of a site is different between the two subfamilies. **A**) non-TIR-NBS subfamily, **B**) TIR-NBS subfamily and **C**) *Q*_k _values of 13 amino acid sites. The threshold (%) for shading was set at 50. Similar amino acid residues are shaded grey, and identical amino acid residues are shaded black.

We also found that the degree of conservation of critical amino acid residues differed between non-TIR- and TIR-NBS-LRR RGAs. For example, the amino acid residue at site 22 was valine (V) and leucine (L) in the non-TIR- and TIR-NBS-LRR RGAs, respectively. Nevertheless, some amino acid residues were highly conserved between the subfamilies, such as those at sites 84 (E), 133 (F) and 155 (W) from the non-TIR-NBS-LRR subfamily and sites 23 (S), 116 (W), 118 (G) and 146 (V) from the TIR-NBS-LRR subfamily (Figure 4). These results suggest that these critical amino acid residue sites are subjected to strong functional constraints within the non-TIR- and TIR-NBS-LRR subfamilies.

### Comparing evolutionary rates among NBS-LRR RGAs in pepper

The evolutionary rates were compared within the non-TIR- and TIR-NBS-LRR subfamilies. The Ka/Ks ratio is an indicator for comparing evolutionary rates in R genes [46]. Generally, a Ka/Ks ratio > 1 implies positive or diversifying selection (i.e. advantageous mutations have been accumulated during the course of evolution); a Ka/Ks ratio < 1 indicates a purifying or negative selection (i.e. most of the non-synonymous substitutions have been eliminated); and a Ka/Ks ratio = 1 indicates neutral selection [47]. In the current paper, we found that within the non-TIR-NBS-LRR subfamily, most Ka/Ks values were <1 in pairwise comparisons, with the exception of CaRGA III/CaRGAIV. This finding suggests that these gene pairs may be under purifying selection. However, CaRGA III/CaRGA IV was under positive selection. Within the TIR-NBS-LRR subfamily, all the Ka/Ks values were <1, indicating that both subfamilies were under strong selective constraints (Table 4). Purifying selection could play a critical role in the evolutionary processes of non-TIR- and TIR-NBS-LRR RGAs in pepper.

**Table 4:** Ka/Ks ratios for pairwise comparisons among members of the non-TIR and TIR-NBS CaRGA subfamilies in pepper

**Group**	**Pairwise comparison**	**Ka**	**Ks**	**Ka/Ks**
Non-TIR-NBS	CaRGA I/CaRGA II	0.218	0.370	0.589
	CaRGA I/CaRGA III	0.571	0.772	0.740
	CaRGA I/CaRGA IV	0.674	0.798	0.845
	CaRGA II/CaRGA III	0.612	0.735	0.833
	CaRGA II/CaRGA IV	0.767	2.441	0.314
	CaRGA III/CaRGA IV	0.656	0.607	1.081
TIR-NBS	CaRGA V/CaRGA VI	0.479	0.823	0.582
	CaRGA V/CaRGA VII	0.462	1.271	0.363
	CaRGA VI/CaRGA VII	0.274	0.718	0.382

### Sliding window analysis

**Figure 5:**
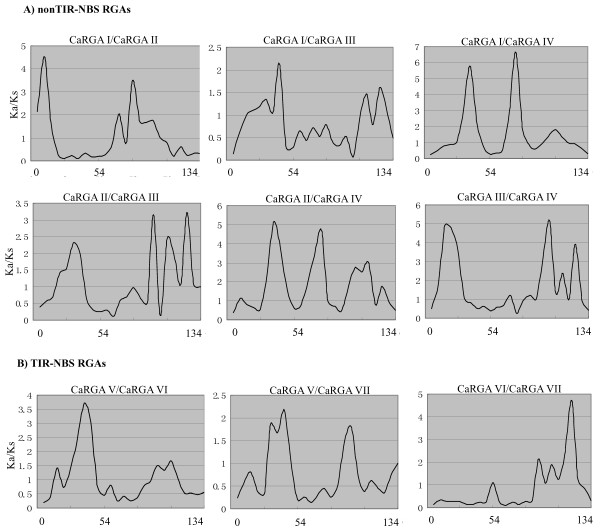
**Sliding window analyses of Ka/Ks ratios for any two groups in the non-TIR- and TIR-NBS subfamilies. **The Y-axis shows the Ka/Ks ratio, and the X-axis shows the amino acid numbers of the aligned sequences.

A sliding window analysis of the Ka/Ks ratios was carried out alongside the determination of the nucleotide sequences of the RGAs for pairwise comparison of any two groups using CRANN software to explore important regions that may have contributed to the functional diversification of NBS-LRR RGAs in pepper [48]. Within the non-TIR-NBS-LRR subfamily, we observed two groups of pairwise comparisons that showed similar tendencies. One was CaRGA I/CaRGA IV and CaRGA II/CaRGA IV, the other was CaRGA II/CaRGA III and CaRGA III/CaRGA IV. The remaining two groups showed different patterns. Overall, the sliding window profile revealed at least three regions of high peaks that were under positive selection, given that all Ka/Ks values were >1 (Figure 5A). Within the TIR-NBS-LRR subfamily, we observed 2–3 regions of high peaks with high Ka/Ks values, consistent with positive selection in three pairwise comparisons (Figure 5B). These regions may be exposed to strong functional constraints. Two of these regions may be located in the RNBS-B and RNBS-C domains, respectively. Therefore, we inferred that the observed difference in the regions of functional constraint may reflect the functional specificities of NBS-LRR RGAs in pepper.

### Determination of functional specificity positions among pepper NBS-LRR RGAs

In plants, many protein families contain homologous proteins that have common biological functions but different specificities towards substrates, ligands, effectors, DNA, proteins and other interacting molecules, including other monomers of the same protein. All these interactions must be highly specific [49]. Thus, important amino acid residues that account for the functional specificity of proteins from a family need to be identified. These important amino acid residue positions are well conserved within specificity groups but differ between different functional subgroups. Such positions are called SDPs (specificity-determining positions) [49]. The SDPfox server was used to predict potential SDPs and identify amino acid residues that are remarkably responsible for the functional specificity of the non-TIR- and TIR-NBS-LRR RGAs of pepper (Table 5 and Additional file 4). Four SDPs (positions 91, 98, 146 and 124) were predicted to be involved in functional specificity. Further analysis revealed that position 98 was located between the kinase-2 and RNBS-B domains. However, currently, there is no function associated with this region. The remaining three SPDs were located in three different conserved domains (i.e. kinase-2, RNBS-B and RNBS-C).

**Table 5:** Predicted specificity-determining residues of the non-TIR and TIR-NBS-LRR RGAs subfamilies in pepper

**No.**	Alignment position*	**R gene group**		**Z-score**	P-value**
		**Non-TIR**	**TIR**		
1	91	W	D	4.72	2.41 × 10^−4^
2	98	W	Y	4.71	2.83 × 10^−8^
3	146	S	A	4.68	3.49 × 10^−12^
4	124	V	L	4.67	3.01 × 10^−16^

*Alignment position designates the amino acid position in the multiple sequence alignment presented in Additional file 3.

**P-value was used to evaluate the significance of the Z-score and assess whether the observed Z-score is sufficiently high to indicate an SDP.

### Expression analysis of CaRGAs in different organs and in response to defence signaling molecules

**Figure 6:**
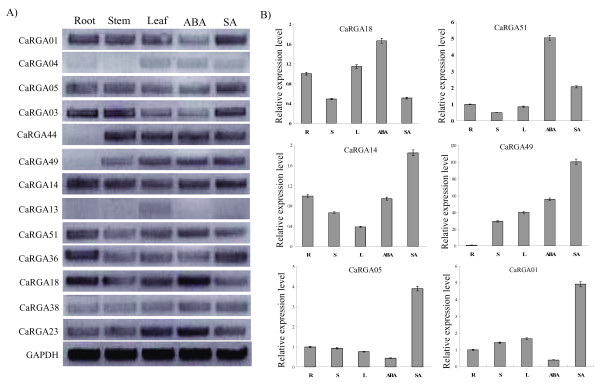
**Expression profiles of 13 pepper NBS-LRR CaRGAs. A**) Expression patterns obtained from semi-quantitative reverse transcriptase PCR experiments in different tissues and in response to the exogenous application of hormones. **B**) Relative transcript levels obtained from quantitative real-time PCR experiments in different tissues and in response to the exogenous application of hormones. Representative images are shown. R: Root, S: Stem and L: Leaf. Glyceraldehyde-3-phosphate dehydrogenase (GAPDH) was used as a reference gene for the expression analysis of pepper CaRGA genes [65].

To analyze the expression levels of CaRGAs in the different organs of pepper, two representatives of each class were randomly used for expression analysis using reverse transcription (RT)-PCR. CaRGA14 and CaRGA15 were the only two members of Class CaRGAIV. Given that the sequence similarity among these RGAs was high, only one pair of specific primers was designed using Primer 5.0 software. A total of thirteen pairs of specific primers were obtained. As shown in Figure 6A, thirteen CaRGAs were expressed in different plant organs, but their expression levels were different. Among these, CaRGA04, CaRGA13 and CaRGA38 were expressed in the leaves, stems and roots at low levels, whereas CaRGA01, CaRGA03, CaRGA14 and CaRGA18 were expressed at relatively high levels. The remaining CaRGAs were expressed at intermediate levels. No expression of CaRGA44 and CaRGA49 genes was detected in the roots. The CaRGAV and II subgroups showed similar expression patterns.

After treatment with abscisic acid (ABA) and salicylic acid (SA), the expression levels of most of these CaRGA genes changed. We found that the addition of ABA increased the transcription levels of several of the analysed CaRGA genes, namely CaRGA18, CaRGA51, CaRGA23, CaRGA14 and CaRGA49. By contrast, ABA addition decreased the expression levels of CaRGA13, CaRGA01 and CaRGA05. However, the expression of CaRGA03, CaRGA36 and CaRGA38 were unchanged. SA is known to play a vital role in plant defence against pathogens [50]. SA also induces the expression of a range of pathogen defence genes in plants [51,52]. Among the CaRGAs tested, CaRGA01, CaRGA05, CaRGA03, CaRGA49, CaRGA14, CaRGA51, CaRGA36 and CaRGA38 displayed the most marked responses to SA treatment (Figure 6A). The expression levels of CaRGA18 and CaRGA23 decreased. The remaining genes showed no response to the SA treatment conditions. Real time-PCR experiments confirmed the expression levels of the selected CaRGAs (Figure 6B).

We found that two members from a subgroup may have different expression patterns, such as CaRGA01 and CaRGA04. However, similar expression patterns were also observed (CaRGA44 and CaRGA49). We also found that the CaRGAIV and VI subgroups had similar expression patterns. However, whether these genes with similar expression patterns have similar functions remains unclear. In addition, some earlier studies have reported that signaling molecules not only function as a critical signal for downstream resistance events but also upregulate the expression of R genes [51-56]. Some CaRGA genes were activated by SA and ABA (Figures 6A and 6B). This suggests that these stimuli induce the expression of the CaRGA genes and that they may play a potential role in mediating cross-talk between signaling pathways.

In summary, this paper provided detailed characterization and data on the functional divergence of non-TIR- and TIR-NBS-LRR CaRGAs in pepper. The mode of selection (positive selection, purifying selection and neutral selection) among the non-TIR- and TIR-NBS-LRR CaRGA subfamilies was identified by Ka/Ks analysis. However, the kind of evolutionary mechanisms responsible for the evolution of R genes in pepper cannot be inferred with certainty without the complete set of NBS-LRR genes from the pepper genome. Future studies must focus on verifying and elucidating the biological function of these CaRGA genes using supplementary experimental approaches, particularly with virus- or *Agrobacterium*-mediated transient assays [57] or by performing loss-of-function experiments, such as virus-induced gene silencing [58].

## Conclusion

The present study identified numerous CaRGA sequences through degenerate PCR amplification and database mining. We divided these CaRGA sequences into two subfamilies (non-TIR- and TIR-NBS-LRR) based on phylogenetic tree and sequence analyses. The identified CaRGAs are a valuable resource for discovering R genes and developing RGA molecular markers that can be used for genetic mapping in pepper. We also predicted thirteen sites (positions 21, 22, 23, 69, 84, 104, 113, 116, 118, 125, 146, 155 and 165) as critical amino acid residues associated with the type-I functional divergence between non-TIR- and TIR-NBS-LRR subfamilies. Ka and Ks analyses showed that a purifying selection could play a critical role in the evolutionary processes of non-TIR- and TIR-NBS-LRR CaRGAs in pepper.

In addition, four SDPs (positions 91, 98, 146 and 124) were predicted to be involved in functional specificity in the non-TIR- and TIR-NBS-LRR CaRGA subfamilies. Expression analysis showed that some CaRGA genes were induced by SA or ABA, suggesting that they may be mainly involved in defence responses activated by signaling pathways associated with these two molecules. These findings provide a better understanding of the evolutionary mechanisms driving the functional diversification of non-TIR- and TIR-NBS-LRR R genes in pepper.

## Methods

### Plant material

The sweet pepper breeding line PBC631B was selected to isolate potential NBS-type disease R genes. PBC631B seeds were germinated, and the seedlings were grown in growth chambers at 25°C for 12 h (day) and 18°C for 12 h (night). Relative humidity was maintained at 65–75%. Young leaves were harvested from 4 week-old plants, immediately frozen in liquid nitrogen and then stored at −80°C for nucleic acid extraction. For hormone treatments, the seedlings were cultured in Hoagland’s solution containing 100 μM SA and 100 μM ABA for 6 h. The treated samples were then harvested for testing. Genomic DNA was isolated using a commercial plant DNA extraction kit (Bioteke, Beijing, China) according to the manufacturer’s instructions.

### Degenerate primers and PCR amplification

**Figure 7:**
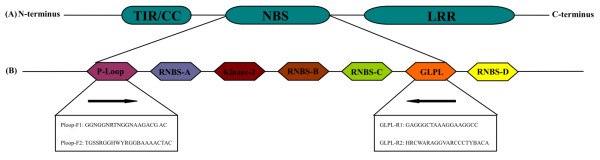
**Schematic representation of plant NBS-LRR R gene structure. **(**A**) Characteristic plant R gene domains**. **(**B**) Conserved motifs within the NBS domain. The solid arrow indicates the position and direction of the designed degenerate forward and reverse primers (P-loop and GLPL conserved motifs, respectively). The degenerate primer sequences are shown in Additional file 5.

Two pairs of degenerate primers were selected for isolating the RGA sequences in pepper (Additional file 5). The positions of the degenerate primers were identified in the two conserved domains (P-loop and GLPL, respectively) in the plant R genes (Figure 7). PCR amplifications were performed using a PTC-100 thermal cycler. PCR amplification reactions were conducted in a total volume of 25 μL containing 20 ng of template DNA, 2 μL of 10× PCR Buffer, 1.5 μL of 25 mmol MgCl_2_, 2 μL of dNTPs (2 mmol/L), 1 μL of Primer-F (10 μmol/L), 1 μL of Primer-R (10 μmol/L), 1 unit of ExTaq DNA (5 U/μL) and 15.3 μL of ddH_2_O. The PCR reaction cycle was as follows: denaturation at 94°C for 4 min, followed by 35 cycles of denaturation at 94°C for 30 s, annealing at 55°C for 30 s and extension at 72°C for 60 s, followed by a final extension at 72°C for 5 min.

### Cloning and sequencing of PCR products

Full volumes of the PCR products were run on a 1.0% agarose gel. Bands of the expected size (~500 bp) were excised from the agarose gel and purified using a DNA gel purification kit (Sangon, Shanghai, China). The obtained DNA was cloned into a pGEM T-Easy vector (Promega, Madison, WI, USA) and transformed into competent *Escherichia coli* JM 10^9^ cells according to the manufacturer’s instructions. The cloned DNA fragment was sequenced by Bio-Asia Company (China).

### Collection of other NBS-LRR RGAs through database mining

Other NBS-LRR RGAs from pepper were identified in the NCBI non-redundant protein database (http://www.ncbi.nlm.nih.gov/) using the key word ‘*Capsicum* resistance genes’. A total of 54 NBS-LRR-type sequences were identified. Six known disease R genes, including *RPM1* (X87851), *Gpa2* (AF195939), *L6* (U27081), *M* (U73916), *N* (U15605) and *Prf* (U65391) were downloaded from the GenBank database, and their phylogenetic relationship with pepper NBS-LRR disease R genes was determined.

### Sequence analysis and phylogenetic tree construction

Each of the acquired DNA sequences was trimmed of vector sequence contamination using VecScreen at NCBI. Identity and similarity searches of nucleotide and amino acid sequences were performed using BLAST at the NCBI GenBank database (http://www.ncbi.nlm.nih.gov/BLAST/). Sequence alignments were carried out using Clustal W (BioEdit software) [59]. The phylogenetic tree was constructed by the NJ method using MEGA 5.0 software [43]. The reliability of the interior nodes was assessed using 1000 bootstrap replicates. Human apoptosis activating factor-1 (Apaf-1), which contains homologous motifs with the NBS region in plant disease resistance genes, was included in the phylogenetic analysis as an outgroup sequence [60].

### Analysis of functional divergence

The phylogenetic tree of the pepper NBS-LRR RGAs was broadly grouped into two clusters, namely the TIR- and non-TIR-NBS-LRR subfamilies. DIVERGE 2 software [61] was used to evaluate the potential functional divergence and to predict the important amino acid residues in these two subfamilies. The coefficients of type I and II functional divergence (*θ*_I_ and *θ*_II_) between these two groups of pepper NBS-LRR RGAs were estimated through posterior analysis. A *θ*_I_ or *θ*_II_ value significantly >1 indicates altered selective constraints or a radical shift in amino acid physiochemical properties after gene duplication and/or speciation [44,45]. A site-specific posterior analysis (*Q*_*k*_) was also used to predict amino acid residues important for functional divergence.

### Calculation of Ka/Ks ratios

We detected the mode of selection (positive selection, purifying selection or neutral selection) among the non-TIR- and TIR-NBS-LRR RGA subfamilies. The Ka/Ks ratios were calculated according to Nei and Gojobori [62] using K-Estimator 6.0 software [63,64]. We identified the NBS-LRR RGAs subject to different selection pressures. DnaSP 5.0 software [65], which can calculate pairwise distance as part of a sliding window analysis, was applied. Ka/Ks values were plotted using Microsoft Excel to produce a graph of Ka and Ks values. The resultant Ka and Ks values were the sum of every possible pairwise comparison between every subgroup of R gene candidates selected for that particular window.

### Analysis of SDPs

SDPfox was used to predict the SDPs that may determine the functional specificity of homologous proteins [49]. SDP presents the statistical significance of the predictions in the form of Z-scores (the number of standard deviations away from the expected value) and displays the most significant positions in a multiple sequence alignment. Positions with high Z-scores are predicted to determine functional specificity.

### RNA isolation, DNase l treatment, cDNA synthesis and semi-quantitative RT-PCR analysis

Total RNA from all samples was isolated using TRIZOL reagent according to the manufacturer’s protocol (Invitrogen). RNA integrity, RNA concentration, RNA quality, DNase l treatment and cDNA synthesis were performed as previously described [66]. Two representatives of each class of NBS-LRR R genes were selected for expression analysis. Gene-specific primer pairs were designed using Primer5.0 software. A total of 13 pairs of CaRGA-specific primers were obtained (Additional file 5). Nine CaRGAs (i.e. CaRGA23, CaRGA38, CaRGA18, CaRGA36, CaRGA51, CaRGA13, CaRGA14, CaRGA49 and CaRGA44) were selected from NCBI. The remaining four CaRGAs (i.e. CaRGA03, CaRGA05, CaRGA04 and CaRGA01) were selected from PCR amplification. Subsequently, we analyzed the visualization of amplicon fragments to verify whether these primers were specific. Primers that exhibited the electrophoresis pattern of a single amplicon with the correct predicted size were considered CaRGA-specific primers. RT-PCR reactions were carried out using an Eppendorf PCR system 5331 cycler. The cycling program was as follows: 10 min at 94°C, 30 cycles of 45 s at 94°C, 45 s at 55°C and 1 min at 72°C and a 7 min extension at 72°C. The glyceraldehyde-3-phosphate dehydrogenase (GAPDH) gene was used as a reference [66].

### Quantitative RT-PCR and data analyses

Primer specificity for quantitative RT-PCR was further confirmed by analyzing melting curves. Primers corresponding to the melting curves that yielded single sharp peaks were used for quantitative RT-PCR analysis. Real-time PCR reactions were carried out in a total volume of 25 μL containing 12.5 μL of 2× SYBRGreen PCR MasterMix (Applied Biosystems), 1 μL of each primer, 1 μL of template (10× diluted cDNA from samples) and 9.5 μL of sterile distilled water. The thermal conditions were as follows: 95°C for 10 min, followed by 40 cycles at 95°C for 15 s and a final step at 60°C for 1 min. Quantification analysis was performed through the comparative CT method. All reactions were performed in triplicate in 96-well reaction plates using an iQ5 machine (Bio-Rad). Two independent replicates were performed. GAPDH was used as a reference gene for the expression analysis of the pepper CaRGA genes [66].

## Competing interests

The authors declare that they have no competing interests.

## Authors’ contributions

HJW and WY participated in conceiving the paper, primer design, sequence analysis, and drafting the final manuscript. MYR and QJY participated in DNA extraction and PCR amplification. RQW, ZML and SJL participated in bioinformatics, and modified the final manuscript. JZ, GZZ and ZPY participated in conceiving the study, and modified the final manuscript. YJY critically reviewed the manuscript and gave financial support to the study. All authors read and approved the final manuscript.

## Supplementary Material

Additional file 1 **PCR amplification products generated by two pairs of degenerate primers in pepper. **Lanes A and B were products of the primer combinations Ploop-1 and GLPL-1 and Ploop-2 and GLPL-2, respectively; M: marker 2000. Click here for file

Additional file 2 DNA and protein sequences of the CaRGAs in pepper. Click here for file

Additional file 3 **Site-specific profile of predicted critical amino acid residues responsible for the functional divergence between the non-TIR and TIR-NBS RGA subfamilies, measured at each site using the posterior probability of being associated with functional divergence. **The arrows point to 13 amino acid residues at which functional divergence between the two subfamilies was predicted. Click here for file

Additional file 4 **Illustration of specificity-determining positions (SDPs) in the non-TIR-and TIR-NBS-LRR subfamilies in pepper. **‘−’ indicates gaps in the alignment. Possible SDPs that might determine functional specificity are highlighted in red and indicated by arrows. Click here for file

Additional file 5 **(A) Degenerate primer sequences used in DNA amplification of NBS-LRR CaRGAs from pepper. **(B) qRT-PCR amplification was used to determine the expression profiles of cloned CaRGAs using the corresponding CaRGA-specific primers. Click here for file
