# Supplementary material for: Analysis of TIR- and non-TIR-NBS-LRR disease resistance gene analogous in pepper: characterization, genetic variation, functional divergence and expression patterns
Source: BMC Genomics. 2012 Sep 21;13:502. doi: 10.1186/1471-2164-13-502 (PMC3472223; doi:10.1186/1471-2164-13-502)
Supplement: Additional file 1 — PCR amplification products generated by two pairs of degenerate primers in pepper. Lanes A and B were products of the primer combinations Ploop-1 and GLPL-1 and Ploop-2 and GLPL-2, respectively; M: marker 2000. [file 1471-2164-13-502-S1.doc]

**Additional file 1** PCR amplification products generated by two pairs of degenerate primers in pepper. Lanes A and B were products of primer combinations of Ploop-F1 and GLPL-R1, Ploop-F2 and GLPL-R2, respectively; M: marker 2000.


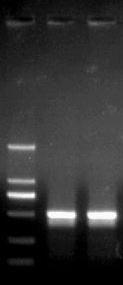


500bp

**M A B**

250bp

100bp

750bp

1000bp

2000bp
