# Supplementary figures and images for: Analysis of TIR- and non-TIR-NBS-LRR disease resistance gene analogous in pepper: characterization, genetic variation, functional divergence and expression patterns
Source: BMC Genomics. 2012 Sep 21;13:502. doi: 10.1186/1471-2164-13-502 (PMC3472223; doi:10.1186/1471-2164-13-502)

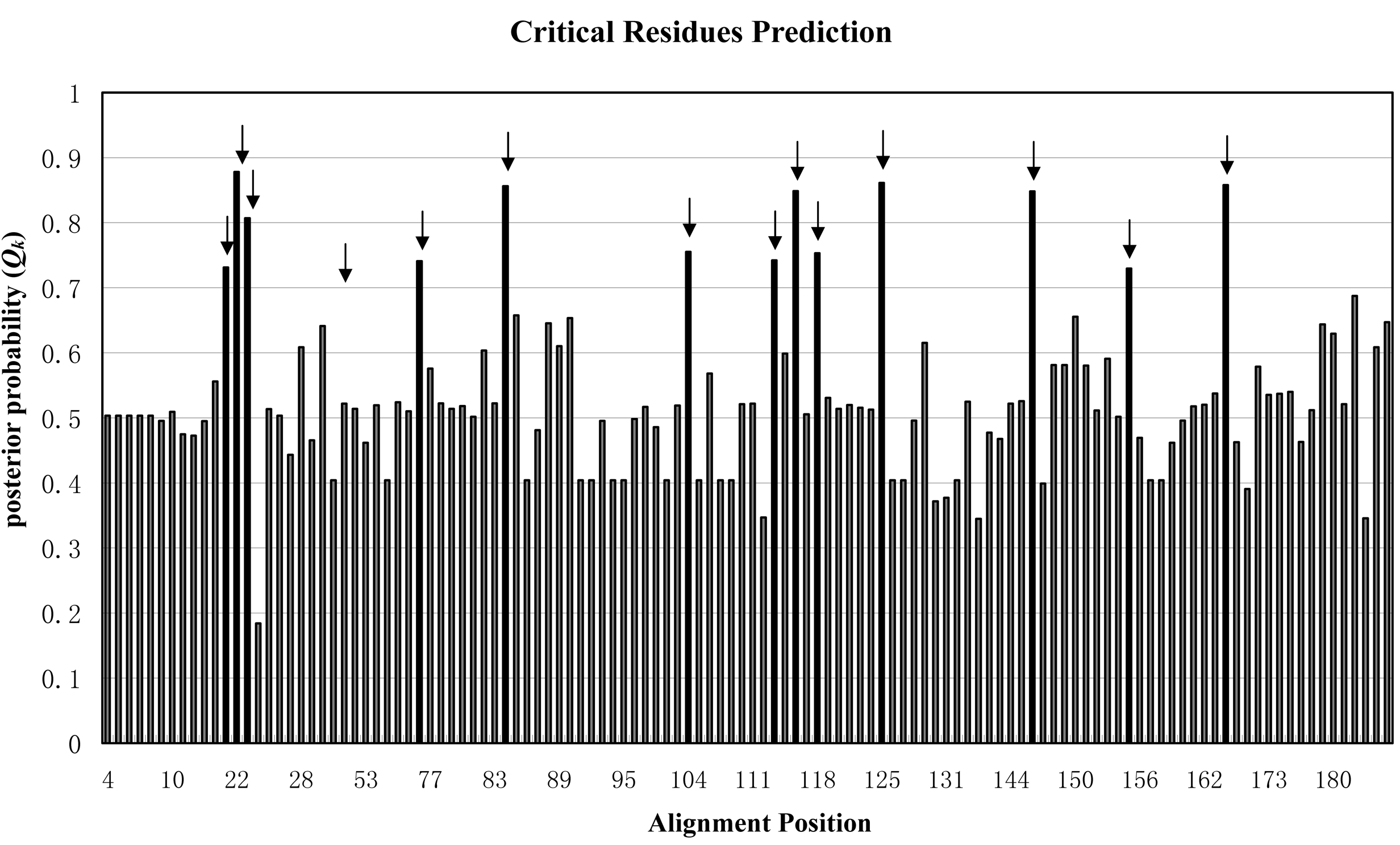

Supplement: Additional file 3 — Site-specific profile of predicted critical amino acid residues responsible for the functional divergence between the non-TIR and TIR-NBS RGA subfamilies, measured at each site using the posterior probability of being associated with functional divergence. The arrows point to 13 amino acid residues at which functional divergence between the two subfamilies was predicted. [file 1471-2164-13-502-S3.tiff]
