# Supplementary material for: Analysis of TIR- and non-TIR-NBS-LRR disease resistance gene analogous in pepper: characterization, genetic variation, functional divergence and expression patterns
Source: BMC Genomics. 2012 Sep 21;13:502. doi: 10.1186/1471-2164-13-502 (PMC3472223; doi:10.1186/1471-2164-13-502)
Supplement: Additional file 4 — Illustration of specificity-determining positions (SDPs) in the non-TIR-and TIR-NBS-LRR subfamilies in pepper. ‘−’ indicates gaps in the alignment. Possible SDPs that might determine functional specificity are highlighted in red and indicated by arrows. [file 1471-2164-13-502-S4.pdf]

```
                    10        20        30        40        50        60        70        80        90       100
           ....|....|....|....|....|....|....|....|....|....|....|....|....|....|....|....|....|....|....|....|
NonTIR-NBS-LRR
CaRGA14  RGSGKTTIAQKIYHDRQVNVRFQKKVWVSISQTYDELS------IMKGILK--QLSADDSGTDKG----DLLNRIREALSRKSYLIVMDDVWSIDDGWWD
CaRGA15  RGSGKTTIAQKIYHDRQVNVRFQKKVWVSISQTYDELS------IMKGILK--QLSADDIGTDKG----DLLNRIREALSRKSYLIVMDDVWSIDDGWWD
CaRGA13  GGLGKTTLAYKVYNDESVCSHFDVRAWFTVDQEYDEKK------LLVKLFN--QVTGSDLKSSED---IDVADKLRKQLYGKRYLIVLDDVWDTT--TWD
CaRGA17  AGSGKTTLAYKVYNDESVCSHFDVRAWFTVDQEYDEKK------LLVKLFN--QVTGSDLKFSED---IDVADKLRKQLYGKRYLIVLDDVWDTT--TWD
CaRGA16  AGLGKTTLAYRVYNDKSVVGHFDVRAWCTVDQERNEKK------LLQRIFN--QVIGLKGSFNEDGIDNDVADKLRKHLIGKRYLIVLDDLWDTA--TWD
CaRGA51  GGVGKTTLARQVYDDIYMEHHFYIRAWITVSQMHQHRE------MLLGILRCFSLVNDNTYLKST---EQLAEQVYRSLKGRRYLIAMDDVWDTS--AWD
CaRGA20  GGVGKTTLAKIVYNDKKVKDHFDLKAWFCVSEAYDSFR------ITKGLLQ--EIGSFDLKDDNN--LNQLQVKLKESLKGKRFLVVLDDLWNDDCDEWD
CaRGA25  GGVGKTTLAKIVYNDKKVKDHFDLKAWFCVSEAYDSFR------ITKGLLQ--EIGSFDLKDDNN--LNQLQVKLKESLKGKRFLVVLDDLWNDDCDEWD
CaRGA34  GGVGKTTLAKIVYNDKKVKDHFDLKAWFCVSEAYDSFR------ITKGLLQ--EIGSFDLKDDNN--LNQLQVKLKESLKGKRFLVVLDDLWNDDCDEWD
CaRGA39  GGVGKTTLAKIVYNDKKVKDHFDLKAWFCVSEAYDSFR------ITKGLLQ--EIGSFDLKDDNN--LNQLQVKLKESLKGKRFLVVLDDLWNDDCDEWD
CaRGA32  GGVGKTTLAKIVYNDKKVKDHFDLKAWFCVSEAYDSFR------ITKGLLQ--EIGSFDLKDDNN--LNQLQVKLKESLKGKRFLVVLDDLWNDDCDEWD
CaRGA23  GGVGKTTLAKIVYNDKKVKDHFDLKAWFCVSEAYDSFR------ITKGLLQ--EIGSFDLKDDNN--LNQLQVKLKESLKGKRFLVVLDDLWNDDCDEWD
CaRGA28  GGVGKTTLAKIVYNDKKVKDHFDLKAWFCVSEAYDSFG------ITKGLLQ--EIGSFDLKDDNN--LNQLQVKLKESLKGKRFLVVLDDLWNDDCDEWD
CaRGA43  GGVGKTTLAKIVYNDKKVKDHFDLKAWFCVSEAYDSFR------ITKGLLQ--EIGSFDLKDDNN--LNQLQVKLKESLKGKRFLVVLDDLWNDDCDEWD
CaRGA21  GGVGKTTLAKIVYNDKKVKDHFDLKAWFCVSEAYDSFR------ITKGLLQ--EIGSFDLKDDNN--LNQLQVKLKESLKGKRFLVVLDDLWNDDCDEWD
CaRGA26  GGVGKTTLAKIVYNDQKVKDHFGLKAWFCVSEAYDAFR------ITKGLLQ--EVGSFDLKDDNN--LNQLQVKLKESLKGKRFLVVLDDVWNDDSNEWD
CaRGA31  GGVGKTTLAKIVYNDQKVKDHFGLKAWFCVSEAYDAFR------ITKGLLQ--EVGSFDLKDDNN--LNQLQVKLRESLKGKRFLVVLDDVWNDDSNEWD
CaRGA19  GGVGKTTLAKIVYNDKKVTGHFGLKAWFCVSEAYDAFR------ITKGLLQ--EIGSFDLKDDNN--LNQLQVKLKESLKGKRFLVVLDGLWNDDSNEWD
CaRGA24  GGVGKTTLAKIVYNDKKVTGHFGLKAWFCVSEAYDAFR------ITKGLLQ--EIGSFDLKDDNN--LNQLQVKLKESLKGKRFLVVLDDLWNDDSNEWD
CaRGA37  GGVGKTTLAKIVCNDKKVTGHFGLKAWFCVSEAYDAFR------ITKGLLQ--EIGSFDLKDDNN--LNQLQVKLKESLKGKRFLVVLDDLWNDDSNEWD
CaRGA41  GGVGKTTLAKAAYNDEKVKSYFNLKAWFCVSEPYDAIR------ITKALLQ--EIGSFDLKDDNN--LNQLQVKLKESLKGKRFLIVLDDMWNDDYNEWN
CaRGA48  -GVGKTTLAKAAYNDEKVIDHFDLKGWFCVSEAYDALR------IAKGLLQ--EIGSFDLNVDDN--LNQLQVKLKEKLNGKRFLIVLDDVWNDNYREWD
CaRGA45  -GVGKTTLAKAVYNDEKVKDHFGLKAWICVSESYDSVR------ITKEPLQ--EISSFDRMVKNT--LNQLQIKLKESLKGKKFLIVLDDVWNDNYEEWD
CaRGA46  --VGKTTLAKAVYNDEKVKDHFGLKAWICVSESYDSVR------ITKELLQ--EISSFDCMVKNT--LNQLQIKLKESLKGKKFLIVLDDVWNDNYEEWD
CaRGA18  GGVGKTTLGKAAYNDEKVQSHFNLTAWFCVSEPYDAFR------ITKGLLQ--QIGS--LQVDDN--LNQLQVKLKESLKGKRFLIVLDDMWNENYNEWD
CaRGA30  GGVGKTTLGKAAYNDEKVQSHFNLTAWFCVSEPYDAFR------ITKGLLQ--QIGS--LQVDDN--LNQLQVKLKESLKGKRFLIVLDDMWNENYNEWN
CaRGA47  -GVGKTTLAKAAYNDENVLSHFNLTAWFCVSEPYDAFR------ITKGLLQ--EIGS--LQVDDN--LNQLQVKLKEILKGKRFLIVLDDMWNENYNEWS
CaRGA22  GGLGKTTLAQAAYNDKKVTNHFKLKAWICVSETYDAIR------ITKGLLQ--EIGSSNSKVDDN--LNQMQVKLKERLKGKMFLIVLDDMWNETYDQWT
CaRGA27  GGLGKTTLAQAAYNDKKVTNHFKLKAWICVSETYDAIR------ITKGLLQ--EIGSSNSKVDDN--LNQMQVKLKERLKGKMFLIVLDDMWNETYDQWT
CaRGA33  GGLGKTTLAQAAYNDKKVSNHFKLKAWICVSETYDAIR------ITKGLLQ--EIGSSNSKVDDN--LNQMQVKLKERLKGKMFLIVLDDMWNETYDQWT
CaRGA35  GGLGKTTLAPAAYNDKKVTNHFKLKAWICVSETYDAIR------ITKGLLQ--EIGSSNSKVDDN--LNQMQVKLKERLKGKMFLIVLDDMWNETYDQWT
CaRGA36  GGMGKTTLAQVVYNYKKVTDHFDLKGWFCVSEAYDASR------ITKALLQ--EIGSIDKKVDDN--LNQLQVKLKERLNGKRFLIVLDDMWNENYREWN
CaRGA42  GGMGKTTLAQVVYNYKKVTDHFDLKGWFCVSEAYDASR------ITKALLQ--EIGSIDKKVDDN--LNQLQVKLKERLNGKRFLIVLDDMWNENYREWN
CaRGA40  GGMGKTTLAQVVYNYKKVTDHFDSKGWFCVSEAYDASR------ITKALLQ--EIGSIDKKVDDN--LNQLQVKLKERLNGKRFLIVLDDMWNENYREWN
CaRGA29  GGMGKTTLAQVVYNYKKVTDHFDLKGWFCVSEAYDASR------ITKALLQ--EIGSFDSKVDDN--LNQLQVKLKERLNGKRFLIVLDDMWNENYREWN
CaRGA38  GGMGKTTLAKAAYSDEKVQSHFNLTAWFCVSEPYDACR------ITKGLLQ--DMGSFDLKDDNN--LNRLQVKLKEELNGKRFLIVLDDVWSDNYNEWD
TIR-NBS-LRR
CaRGA01  GGVGKTTIARAIFD--TLSYRFKAACFLADVKEKAKGN---QLHSLQNILLSELLRKKHDYVYNKL---DGKCMIPERLCSMKVLIVLDDIDHIDHLEYL
CaRGA02  GGIGKTTIARAIFD--TLSYRFKAACFLADVKEKAKGN---QLHSLQNILLSELLRKKHDYVYNKL---DGKCMIPERLCSMKVLIVLDDIDHIDHLEYL
CaRGA10  GGIGKTTIARAIFD--TLSYRFKAACFLADVKEKAKGN---QLHSLQNILLSDLLRKKHDYVYNKL---DGKCMIPERLCSMKVLIVLDDIDHIDHLEYL
CaRGA07  GGVGKTTIARAIFD--TLSYRFKAACFLADVKENAKRN---QLHSLQNILLSELLKKKDDYVNNKL---DGKCMIPTRLCSMKVLIVLDDIDHSDHLEYL
CaRGA11  GGVGKTTIARAIFD--TLSYRFKAACFLADVKENTKRN---QLHSLQNILLSELLKKKDDYVNNKL---DGKCMIPTRLCSMKVLIVLGDIDHSDHLEYL
CaRGA08  GGVGKTTIARAIFD--TLSYRFKTACFLADVKENGKKN---QLHSLQNILLSELLKKKDNYINNKL---DGKCMIPTRLCSMKVLIVLDDIDHSDHLVYL
CaRGA12  GGVGKTTIARAIFD--TLSYRFKSACFLADVKENGKKN---QLHSLQNILLSELLKKKDNYINNKL---DGKCMIPTRLCSMKVLIVLDDIDHSDHLEYL
CaRGA04  GGVGKTTIATAIFHKFSLSVQFEAACILDDVKENAKKN---GLCSLQNILLSELLGEKDNYVKSQQ---VGKCMIPSRLCSMKVLIVLDDIDHNEHLEYL
CaRGA03  GGIGKTTIARAVFD--AHSCQFEAACFIEDIKEN--KC---GMRFLQNILLSELLREKDNYVNNK----DGKHMIARRLPFNKVLVVLDDIDHSDHLHYL
CaRGA05  GGVGKTTIARAVFD--AHSCQFEAACFIEDIKEN--KC---GMRFLQNILLSELLREKDNYVNNK----DGKHMIARRLPFNKVLVVLDDIDHSDHLHYL
CaRGA06  GGMGKTTIARAVFD--AHSCQFEAACFIEDIKEN--KC---GMRFLQNILLSELLREKDNYVNNK----DGKHMIARRLPFNKVLVVLDDIDHSDHLHYL
CaRGA09  ANWARRRMLPAAMA--AAGIRLDRPC------------------FLQNILLSELLREKDNYVNNK----DGKHMIARRLPFNKVLVVLDDIDHSDHLHYL
CaRGA49  GGVGKTTIASGIFD--EISSQFEGSCFLANVWSVLKKSELEVLQHLQQKLLSQILKKDSVNVPNFA---TGDEMISQMLRFKKVLIVLDDMDDSQQLEYL
CaRGA50  GGVGKTTIASGIFD--EISSQFEGSCFLANVWSVLKKSELEVLQHLQQKLLSQILKKDSVNVPNFA---TGDEMISQMLRFKKVLIVLDDMDDSQQLEYL
CaRGA44  ---GKTTIAREIFG--VISCQFEGCCFLANVRSVLKKSGLGGLQHLQQKLLSQILKKDSVNVPNFA---RGDEMMSQMLRFKKVLIVLDDVDDSHQLEYL
```

```
                       110       120       130       140       150       160       170       180
                   ....|....|....|....|....|....|....|....|....|....|....|....|....|....|....|..
NonTIR-NBS-LRR
         CaRGA14  RISRGLPKFIEHNSCIIITSRNEDVVKRMGATESRIHRPRLLDDEESWSLFCKVALLSTKGK--CNTQLEEVGREIVRKCQGFPLA-
         CaRGA15  RISRGLPKFIEHNSCIIITSRNEDVVKRMGATESRIHRPRLLDDEESWSLFCKVALLSTKGK--CNTQLEEVGREIVRKCQGFPLA-
         CaRGA13  DLTRPFP-EAEKGSRIILTTRQKEVAFHGKCNTD-PLNLRLLRPEESWELLEKRAFRKESCP----DELLDVGKEIAQNCQGLPLA-
         CaRGA17  DLTRPFP-EAEKGSRIILMTRQKEVAFHGKCNTD-PLNLRLLRPEESWELLEKRAFRKESCP----DELLDVGKEIAQNCQGLPLA-
         CaRGA16  ELTRPFPSEFQKGSRVILTSRKKEVAVHGKYHSD-PLDLRLLRLEESWELLEKRVFGEEHCP----DELKDVGEKIARKCQGLPLA-
         CaRGA51  VVKRSFP-DDKNGSRVILTSRIANVGIYASSGS--PHYMQCLSVEESLKLFNLKAFGRETCP----LELEKATKQIVEKCQGLPLAL
         CaRGA20  DLKNLFV-QGAMGSKILVTTRKEDVALMMGNGA---INVETLSDEVSWDLFKQHSLK-NRDP-EEHPELEEVGKQIADKCKGLPLAL
         CaRGA25  DLKNLFV-QGAMGSKILVTTRKEDVALMMGNGA---INVETLSDEVSWDLFKQHSLK-NRDP-EEHPELEEVGKQIADKCKGLPLAL
         CaRGA34  DLKNLFV-QGAMGSKILVTTRKEDVALMMGNGA---INVETLSDEVSWDLFKQHSLK-NRDP-EEHPELEEVGKQIADKCKGLPLAL
         CaRGA39  DLKNLFV-QGAMGSKILVTTRKEDVALMMGNGA---INVETLSDEVSWDLFKQHSLK-NRDP-EEHPELEEVGKQIADKCKGLPLAL
         CaRGA32  DLKNLFV-QGAMGSKILVTTRKEDVALMMGNGA---INVETLSDEVSWDLFKQHSLK-NRDP-EEHPELEEVGKQIADKCKGLPLAL
         CaRGA23  DLKNLFV-QGAMGSKILVTTRKEDVALMMGNGA---INVETLSDEVSWDLFKQHSLK-NRDP-EEHPXLEEVGKQIADKCKGLPLAL
         CaRGA28  DLKNLFV-QGAMGSKILVTTRKEDVALMMGNGA---INVETLSDEVSWDLFKQHSLK-NRDP-EEHPELEEVGKQIADKCKGLPLAL
         CaRGA43  DLKNLFV-QGAMGSKILVTTRKEDVALMMGNGA---INVETLSDEVSWDLFKQRSLK-NKDP-EEHPELEEVGKQIADKCKGLPLAL
         CaRGA21  DLKNLFV-QGAMGSKILVTTRKEDVALMMGNGA---INVETLSDEVSWDLFKQHSLK-NRDP-EEHPELEEVGKRIADKCKGLPLAL
         CaRGA26  DLRNLFV-EGAMGSKIIVTTRKENVAGMMDSGA---INVGTLSDEASWALFKQHSLK-NRDP-EEHPELEEVGKKIANKCKGLPLAL
         CaRGA31  DLRNLFV-EGAMGSKIIVTTRKENVAGMMDSGA---INVGTLSDEASWALFKQHSLK-NRDP-EEHPELEEVGKKIANKCKGLPLAL
         CaRGA19  DLRNVFV-EGAMGSKIIVTTRKESVALMMGSGE---INVRTLSDEASWALFRRHSLE-NRDPPKEYPELEEVGKQIADKCKGLPLAL
         CaRGA24  DLRNVFV-EGAMGSKIIVTTRKESVALMMGSGE---INVRTLSDEASWALFRRHSLE-NRDPPKEYPELEEVGKQIADKCKGLPLAL
         CaRGA37  DLRNVFV-EGAMGSKIIVTTRKESVALMMGSGE---INVRTLSDEASWALFRRHSLE-NRDPPKEYPELEEVGKQIADKCKGLPLAL
         CaRGA41  DLRTPFV-HGDMGSKIIVTTRKKDVAGMMGSGA---VNVGTLSSEASWALFKRHSLE-NRYP-EEHPELEEVGKQISDKCKGLPLAL
         CaRGA48  DLRNLFV-QGATGSKTIVTTSKVNVAQMIGSGA---INMGILSSEASWALFKRHSLE-NRDPPKEHPELEEVGKQIADKCKGLPLA-
         CaRGA45  DLRNIFV-QGDIGSKIIVTTRKETVSLMMSSRA---INVGTLSSEVSWALFKRHSFE-NRAP-EEHPEFQRGWKANCTQVQRIAIIS
         CaRGA46  DLRNIFV-QGDIGSKIIVTTRKETVSLMMSSRA---INVGTLSSEVSWALFKRHSFE-NRAP-EEHPEFQEVGKQ-LHASARIAIIS
         CaRGA18  DLWNVFV-QGGIGSKIIVTTRKESVALMMQTEQ---ISMDTFSIDDSWSLFKRHAFE-NMDP-MEHPELEEVGKQIVAKCKGLPLAL
         CaRGA30  DLWNVFV-QGGIGSKIIVTTRKESVALMMQTEQ---ISMDTFSIDDSWSLFKRHAFE-NMDP-MEHPELEEVGKQIVAKCKGLPLAL
         CaRGA47  DLWNVFV-QGGIGSKIIVTTRKEGVALMMGSEQ---ISKDTLSIDDSWSLFKRHAFE-NMNP-MGHLEIEEVGKQTASKCQGLPLAL
         CaRGA22  DLRNIFD-QGGIGSTIIVTTRKASVARMMLAEQ---ISMDTLSIDDSWSLFERHAFE-NMDP-KEHPELEKVAKKIAARCKGLPLAL
         CaRGA27  DLRNIFD-QGGIGSTIIVTTRKASVARMMLAEQ---ISMDTLSIDDSWSLFERHAFE-NMDP-KEHPELEKVAKKIAARCKGLPLAL
         CaRGA33  DLRNIFD-QGGIGSTIIVTTRKASVARMMLAEQ---ISMDTLSIDDSWSLFERHAFE-NMDP-KEHPELEKVAKKIAARCKGLPLAL
         CaRGA35  DLRNIFD-QGGIGSTIIVTTRKASVARMMLAEQ---ISMDTLSIDDSWSLFERHAFE-NMDP-KEHPELEKVAKKIAARCKGLPLAL
         CaRGA36  DLRNIFV-QGGIGSKIIVTTRKKSVALMMRAEQ---ISMDTLSIDDSWSLFKRHAFE-NMDP-MGHPELEEVGKRIAAKCKGLPLAL
         CaRGA42  DLRNIFV-QGGIGSKIIVTTRKKSVALMMRAEQ---ISMDTLSIDDSWSLFKRHAFE-NMDP-MGHPELEEVGKRIAAKCKGLPLAL
         CaRGA40  DLRNIFV-QGGIGSKIIVTTRKKSVALMMRAEQ---ISMDTLSIDDSWSLFKRHAFE-NMDP-MGHPELEEVGKRIAAKCKGLPLAL
         CaRGA29  DLRNIFV-QGGIGSKIIVTTRKKSVALMMRAEQ---ISMDTLSIDDSWSLFKRHAFE-NMDP-MGHPELEEVGKRIAAKCKGLPLAL
         CaRGA38  DLRNIFV-HGDIGSKIIVTTRKESVALMIGSGA---IYEGTLSDEDSWALFRRHSLE-NGDP-MGHAELAEVGKQIAAKCKGLPLAL
TIR-NBS-LRR
         CaRGA01  AGDLRWF---GDGSRVVITTRNRQLIE-NDDAI---YEVSTLPDDEAMLLFNQHAFK-NKIP---HESFKMMSLEVLSHAKGLPLAL
         CaRGA02  AGDLRWF---GDGSRVVITTRNRQLIE-NDDAI---YEVSTLPDDEAMLLFNQHAFK-NKIP---HESFKMMSLEVLSHAKGLPLAL
         CaRGA10  AGDLRWF---GDGSRVVITTRNRQLIE-NDDAI---YEVSTLPDDEAMLLFNQHAFK-NKIP---HERFKMMSLEVVSHAKGLPLAL
         CaRGA07  AGDRCWF---GNGSRVVITTRNRQLIE-NDDSV---YEVSTLPDDEAVLLFNQHALK-KKIP---DECCKSFSLKVVNHAKGLPLAL
         CaRGA11  AGDRCWF---GNGSRVVITTRNRQLIE-NDDSV---YEVSTLPDDEAMLLFNQHALK-KKIP---DECCKSFSLKVVNHAKGLPLAL
         CaRGA08  AGGLHWF---GSGSRVVITTRNRQLIE-NADAI---YEVSTLPDDEAMLLFNQHAFK-NKIP---DECFKMFSLEVVNHAKGLPLAL
         CaRGA12  AGGLHWF---GGGSRVVITTRNRQLIE-NADAI---YEVSTLPDDEAMLLFNQHAFK-NKIP---DECFKMFSLEVVNHAKGLPLAL
         CaRGA04  AGDASWF---GKGSRVIVTTTNRDLIKINDDAI---YEVKLLPDREAMQLFNQYAFR-KEAP---DERFKKLSLEAVNHAKGLPLAL
         CaRGA03  AGNLSWY---GNGSRIIATTRDKHLFG-RNDVV---YEVTTLVDRQAIKLFNQYAFK-EEVP---DECFEKLSLEIVRHANGLPLAL
         CaRGA05  AGNLSWY---GNGSRIIATTRDKHLFG-RNDVV---YEVTTLVDRQAIKLFNQYAFK-EEVP---DECFEKLSLEIVRHANGLPLAL
         CaRGA06  AGNLSWY---GNGSRIIATTRDKHLFG-RNDVV---YEVTTLVDRQAIKLFNQYAFK-EEVP---DECFEKLSLEIVRHAKGLPLAL
         CaRGA09  AGNLSWY---GNGSRIIATTRDKHLFG-RNDVV---YEVTTLVDRQAIKLFNQYAFK-EEVP---DECFEKLSLEIVRHANGLPLAL
         CaRGA49  VGKRDWF---GDGTKVITTTTRNLDLLS-KHDVL---YRVPELTNHEALELFSWHAFQ-QGTP---VEGFEELSCCVVDYAKGFPLAL
         CaRGA50  VGKRDWF---GDGTKVITTTTRNLDLLS-KHDVL---YRVPELTNHEALELFSWHAFQ-QGTP---VEGFEELSCCVVDYAKGFPLAL
         CaRGA44  VGKRDWF---GDGTRVLITTRNFDLLS-KHDVL---YRVPELTNHEALELFSWHAFK-QGTP---VDGFEELSYCVVDYA-------
```
