# Supplementary material for: Analysis of TIR- and non-TIR-NBS-LRR disease resistance gene analogous in pepper: characterization, genetic variation, functional divergence and expression patterns
Source: BMC Genomics. 2012 Sep 21;13:502. doi: 10.1186/1471-2164-13-502 (PMC3472223; doi:10.1186/1471-2164-13-502)
Supplement: Additional file 5 — (A) Degenerate primer sequences used in DNA amplification of NBS-LRR CaRGAs from pepper. (B) qRT-PCR amplification was used to determine the expression profiles of cloned CaRGAs using the corresponding CaRGA-specific primers. [file 1471-2164-13-502-S5.doc]

**Additional file 5**

**(A)** Degenerate primers used for PCR amplification from pepper

| Degenerate primer | Primer sequence (5’-3’) | Size (bp) | Conserved domains | Reference |
| --- | --- | --- | --- | --- |
| Ploop-F1 | GGNGGNRTNGGNAAGACG AC | 20 | GG(I/M/V)GKTT | Noir et al. (2001) |
| GLPL-R1 | GAGGGCTAAAGGAAGGCC | 18 | GLPLAL | Deng et al. (2000) |
| Ploop-F2 | TGSSRGGHWYRGGBAAAACTAC | 22 | (A/G/P/R)G(T/I/M/S/L)GKTT | Zhang et al. (2008) |
| GLPL-R2 | HRCWARAGGVARCCCTYBACA | 21 | GLPL(A/T)L |

The sequences are coded according to the International Units of Biochemistry: N = A or C or G or T; R = A or G;

S = C or G; Y = C or T; W= A/T; Y= T/C; B= G/C/T; H=A/T/C; V = A or C or G

Reference:

Deng Z, Huang S, Ling P, Chen C, Yu C, Weber CA, Moore GA, Gmitter FG (2000) Cloning and characterization of NBS-LRR class resistance -gene candidate sequences in citrus. Theor Appl Genet 101: 814-822

Noir S, Combes M-C, Anthony F, Lashermes P (2001) Origin, diversity and evolution of NBS-type disease-resistance gene homologues in coffee trees (Coffea L.). Mol Gen Genomics 265: 654-662

Zhang LY, Chen RG, Zhang JH (2008) Cloning and analysis of resistance gene analogs from pepper (*Capsicum annuum* L.). *Agr Sci China* (in Chinese) 41:169-175.

**(B)** (q)RT-PCR amplification was used to determine the expression profiles of cloned CaRGA using the corresponding CaRGA-specific primers.

| Clone name | Primer code | Sequence forward (5’ 3’) | Sequence reverse (5’ 3’) | Amplicon size (bp) | Temp (℃) |
| --- | --- | --- | --- | --- | --- |
| CaRGA23 | F1/R1 | AAGAGCGATTGATTGACCGT | CCAACAATAGGGACAACAGT | 73 | 58 |
| CaRGA38 | F2/R2 | TTCTGGATGATGTGTGGAGT | ATCATCAAGGCAACACTCTC | 124 | 58 |
| CaRGA18 | F3/R3 | TCGGCAGAATGAAATAGAGG | ACAATAGGGACAACAGCCG | 84 | 58 |
| CaRGA36 | F4/R4 | AGAGTGTTGCCTTGATGATG | TTCCACCCTTCAACCTCTG | 229 | 58 |
| CaRGA51 | F5/R5 | GGAGCATCACTTCTACATTC | CTCTTTAGGTAGGTGTTGTC | 120 | 58 |
| CaRGA13 | F6/R6 | GGTTACTGGCTCGGATTTG | TTCATCAGGGCAACTCTCTT | 310 | 58 |
| CaRGA14 | F7/R7 | GGAGTATTGATGATGGATGG | ATTCTTGATTCTGTGGCTCC | 133 | 58 |
| CaRGA49 | F8/R8 | AGGAGACGAAATGATAAGCC | ATCACCAAACCAATCACGCT | 118 | 58 |
| CaRGA44 | F9/R9 | TTGCCAGAGGAGATGAAATG | AACCAATCACGCTTTCCAAC | 118 | 58 |
| CaRGA03 | F10/R10 | ATAGGGAAGACGACAATAGC | GCAAATGGTCGCTGTGATC | 253 | 58 |
| CaRGA05 | F11/R11 | ATAAGGACGGAAAGCACATG | CTACATCATTCCTCCCAAAC | 183 | 58 |
| CaRGA04 | F12/R12 | GGTAAGACGACAATAGCAAC | CATCACCTGCTAAATACTCC | 283 | 58 |
| CaRGA01 | F13/R13 | ATTTGGAGTATTTGGCAGGC | ACCTCCAACGACATCATCTT | 208 | 58 |
